# Can pay‐for‐performance to primary care providers stimulate appropriate use of antibiotics?

**DOI:** 10.1002/hec.3535

**Published:** 2017-07-07

**Authors:** Lina Maria Ellegård, Jens Dietrichson, Anders Anell

**Affiliations:** ^1^ Department of Economics Lund University Lund Sweden; ^2^ SFI The Danish National Centre for Social Research Copenhagen Denmark; ^3^ Department of Business Administration Lund University Lund Sweden

**Keywords:** antibiotic resistance, pay‐for‐performance, primary care

## Abstract

Antibiotic resistance is a major threat to public health worldwide. As the healthcare sector's use of antibiotics is an important contributor to the development of resistance, it is crucial that physicians only prescribe antibiotics when needed and that they choose narrow‐spectrum antibiotics, which act on fewer bacteria types, when possible. Inappropriate use of antibiotics is nonetheless widespread, not least for respiratory tract infections (RTI), a common reason for antibiotics prescriptions. We examine if pay‐for‐performance (P4P) presents a way to influence primary care physicians' choice of antibiotics. During 2006–2013, 8 Swedish healthcare authorities adopted P4P to make physicians select narrow‐spectrum antibiotics more often in the treatment of children with RTI. Exploiting register data on all purchases of RTI antibiotics in a difference‐in‐differences analysis, we find that P4P significantly increased the share of narrow‐spectrum antibiotics. There are no signs that physicians gamed the system by issuing more prescriptions overall.

## 1 INTRODUCTION

Pay‐for‐performance (P4P), monetary incentives to reach predefined targets, is ubiquitous in health care. Literature reviews conclude that incentives tied to process measures, such as screening rates and guideline adherence, often have small to moderate effects, whereas P4P has no impact on broad outcome measures such as mortality (Christianson, Leatherman, & Sutherland, 2008; Eijkenaar, Emmert, Scheppach, & Schöski, 2013; Ogundeji, Bland, & Sheldon, 2016; Petersen, Woodard, & Urech, 2006; Rosenthal & Frank, 2006; Scott, Sivey & Ait Ouakrim, 2011; Town, Kane, Johnson & Butler, 2005; Van Herck, De Smedt, Annemans, & Remmen, 2010). The reviews also point at gaps in the literature: Many studies lack adequate control groups (Ogundeji et al., 2016), and there are few studies from outside the UK or the United States (Eijkenaar et al., 2013). This study provides evidence from Sweden, where P4P became widely adopted in primary care during the past decade. With one exception, Ödesjö, Anell, Gudbjörnsdottir, Thorn, and Björck (2015) who examine incentives tied to diabetes registry entries in one county, Swedish applications of P4P have not been studied before. We take advantage of the unique institutional setting, in which the responsibility for health care is decentralized to 21 county councils. Due to the decentralization, monetary incentives have been introduced in some counties but not in others, making it possible to evaluate P4P by comparing counties that use specific incentives with counties that do not.

Another contribution of the study is to focus on a scarcely studied P4P application of great policy relevance, namely, incentives related to antibiotic prescriptions. Antimicrobial resistance is one of the greatest threats to the effectiveness of health care (ECDC/EMEA Joint working group, 2009; Carlet et al., 2011). To impede the spread of resistance, physicians ought to engage in antibiotic stewardship: that is, prescribe antibiotics only when necessary and select antibiotics that act against as narrow a spectrum of bacteria as possible (Kaier & Moog, 2012; Ranji, Steinman, & Shojania, 2006). However, inappropriate use of antibiotics is common. 1In the United States, almost half of all prescriptions for acute respiratory tract infections—the most common reason for antibiotics use in the United States—are inappropriate (Fleming‐Dutra, Hersh & Shapiro, 2016).Results for other process measures cannot readily be extrapolated to antibiotics‐related P4P, which imply more than “box‐ticking”: Physicians may bear a real cost from denying patients desired antibiotics (Ashworth et al., 2015). There are only four previous studies of P4P tied to antibiotic stewardship, of which three find positive associations (Gong, Qiu, Song, & Sun, 2016; McDonald, Boaden, Roland, & Kristensen, 2015; Yip, Powell‐Jackson, & Chen, 2014) and one finds a negative relationship (Mullen, Frank, & Rosenthal, 2010).

We study the effect of P4P for antibiotic stewardship in the treatment of children with respiratory tract infections (RTI), introduced by eight counties in a staggered manner. Using municipality‐level register data on all purchases of RTI antibiotics prescribed to Swedish children aged 0–6 years in 2006–2013, we estimate a difference‐in‐differences (DID) model of the impact of P4P. The incentives we study encouraged physicians to select narrow‐spectrum antibiotics in the treatment of children with RTI. Specifically, the incentives were tied to the share of narrow‐spectrum penicillin V (PcV) prescriptions in childrens' total consumption of RTI antibiotics. The focus on the PcV share mirrored Swedish RTI treatment guidelines, which advocate PcV as the first‐line antibiotic in most cases because of its smaller impact on resistance.

The next section gives a background to RTI and Swedish treatment guidelines. Section 3 reviews related literature. Section 4 gives an institutional background and describes the P4P schemes. Section 5 describes the data, Section 6 outlines our empirical strategy, and Section 7 presents the results. Section 8 concludes.

## 2 RTI AND ANTIBIOTICS

Respiratory tract infections is an umbrella term for conditions affecting the respiratory organs, for example, sore throat (pharyngitis), ear infection (otitis), influenza, cough (bronchitis), pneumonia, sinus infection, tonsillitis, laryngitis, and the common cold. RTI is highly associated with antibiotics prescriptions. In Sweden, antibiotics that are typically prescribed for RTI account for 90% of children's total antibiotics consumption (SWEDRES‐SVARM, 2013) and approximately half of all RTI patients in primary care receive antibiotics (André et al., 2008). However, there is sometimes a weak clinical basis for prescribing antibiotics. Many patients seek care for viral RTI, such as the common cold, which are not cured by antibiotics. For bacterical RTI, antibiotics may reduce the symptom spell by a few days but in most cases do not yield large health gains. 2See for example, Spurling, Doust, Del Mar and Eriksson (2011); Lemiengre et al. (2012); Spinks, Glasziou and Del Mar, (2013); Venekamp, Sanders and Glasziou, (2013); Cronin, Khan and Saeed, (2013); Smith, Fahey, Smucny, and Becker, (2014).Nevertheless, some bacterial infections carry a small risk for detrimental complications that require antibiotic treatment. Fear of such consequences, together with diagnostic uncertainty, may explain the widespread use of antibiotics for RTI (e.g., Keith, Saxena, Murray, & Sharland, 2010).

The use of antibiotics gives an evolutionary advantage to resistant bacteria strains (Kaier & Moog, 2012). To impede the spread of resistance, it is advisable to choose an antibiotic that acts against a narrow spectrum of bacteria whenever possible (Hersh, Shapiro, Pavia, & Shah, 2011). According to the Swedish treatment guidelines, narrow‐spectrum antibiotics are often sufficient treatment for patients with bacterial RTI; specifically, PcV is the recommended first‐line antibiotic for most common RTIs. 3Swedish RTI guidelines are available at https://www.folkhalsomyndigheten.se/smittskydd-beredskap/antibiotika-och-antibiotikaresistens/behandling srekommendationer/, last accessed 2016‐12‐06. PcV has long been the most common RTI antibiotic in Sweden (André et al., 2008) but is much less used outside Scandinavia (Goossens et al., 2005, 2007). In 2009, the renowned and government‐supported network Strategic Programme against Antibiotic Resistance (Strama) 4
http://strama.se/about-strama/?lang=en, last accessed 2016‐05‐19.proposed as a national target that PcV should account for 80% of RTI antibiotics prescriptions to children aged 0–6 years. The reason for the focus on children was that most RTI patients in this age group benefit little from antibiotics and that PcV is often sufficient when antibiotics are needed. 5See Online Appendix for English translation of targets.Notably, the target still allowed for broad‐spectrum antibiotics when they were needed.

## 3 RELATED LITERATURE

There is one previous study of P4P in Swedish primary care. Ödesjö et al. (2015) study financial incentives for register entry of diabetes patients introduced in the county Västra Götaland in 2011. They compare the 1‐year development with one other county (Skåne) that did not use this type of P4P and find increased register entry, increased completeness of data, and altered blood pressure entry behavior.

Of the four previous studies addressing incentives for antibiotic stewardship, two Chinese studies find reduced prescription rates (2–7 percentage points, or 15–60%) (Gong et al., 2016; Yip et al., 2014), a British study reports a 2–3 percentage point (3%) improvement of the rate of appropriate antibiotics (McDonald et al., 2015), and a U.S. study finds deteriorations of a similar outcome (Mullen et al., 2010). 6Martens, Werkhoven, Severens, and Winkens (2007) examine an antibiotics‐related financial incentive that was paid ex ante (thus unrelated to performance). They found an advantageous but short‐lasting effect.These studies have notable methodological weaknesses. Only two (Yip et al., 2014; Mullen et al., 2010) have control groups, and one study (McDonald et al., 2015) even lacks a before‐P4P baseline measure. In all four studies, P4P was coupled with other important policy changes—complete restructuring of the payment scheme from fee‐for‐service (FFS) to capitation (Yip et al., 2014), the introduction of other P4P indicators (McDonald et al., 2015; Mullen et al., 2010), and the simultaneous introduction of audit and feedback (Gong et al., 2016).

The literature on non‐financial interventions for antibiotic stewardship suggests that education and audit and feedback have moderate effects (Arnold and Straus, 2005; Ranji et al., 2006; van der Velden, Pijpers, & Kuyvenhoven, 2012; Vodicka, Thompson & Lucas, 2013). 7For recent studies, see Meeker et al. (2014); Meeker, Linder, and Fox (2016); Hallsworth et al. (2016).Notably though, studies in Scandinavian settings typically report insignificant effects (Bjerrum et al., 2011; Munck, Gahrn‐Hansen, Sögaard, & Sögaard, 1999; Ranji et al., 2006). One exception is an educational intervention in Norway, which increased the PcV share from 45% to 54%(20%) (Gjelstad et al., 2013), though part of this may be a Hawthorne effect.

## 4 INSTITUTIONAL BACKGROUND

### 4.1 Organization of primary care

In Sweden, health care is the responsibility of 21 independent county councils. Primary care is typically provided in group practices, with a handful of general practitioners and a team of nurses, social workers and behavioral therapists, physiotherapists, and midwives. Solo practices are very rare (Anell, Glenngård, & Merkur, 2012a). Primary care providers are referred to as primary care centers in this paper. The counties operate most of the about 1,500 primary care centers, though an increasing share (about 40% as of 2013) is private.

In all counties, patients pay visit fees and part of the price for outpatient drugs, 8Patients pay the full price for prescription drugs up to a yearly cap, above which the county subsidizes a progressively larger part. Expenditure above an upper cap (currently about 240 euros per year) are fully subsidized. The caps and subsidy rates for prescription drugs are nationally regulated and thus the same in all county councils, as are pharmacies' prices of subsidized drugs.but income taxes are the dominant source of funding. Each county sets its own tax rate and designs the reimbursement scheme for primary care centers. The reimbursement is a mix of capitation, FFS, and P4P. Capitation is the dominant payment type, accounting for 70–98% of reimbursement in most counties during the sample period (one exception is Stockholm county, where the capitation share was 40%). The remaining part of revenues is predominantly FFS (Anell et al., 2012a). P4P is a complementary reimbursement, which gained in popularity during the past decade: The number of counties using P4P increased from 5 to 20 between 2006 and 2012. Incentives are commonly tied to, for example, prescription targets, patient ratings, registrations in quality registers, and vaccination rates (Anell, Nylinder, & Glenngård, 2012b). In 2012, P4P accounted for 1–5% of total reimbursement. Notably, the P4P reimbursement is determined by the performance of the care center, that is, the payment goes into the care center's budget. Physicians typically earn a fixed salary, and their reimbursement is not directly affected by the P4P incentive.

#### 4.1.1 PcV‐related P4P

To increase PcV's share of RTI antibiotics to children aged 0–6 years, eight counties introduced P4P during our sample period (Table 1). The counties developed their P4P indicators independently but chose similar performance measures, with only minor variations regarding the definition of “other” RTI antibiotics and (in two counties) restrictions of included diagnoses. The indicators were likely inspired by Strama's national PcV target (see Section 2).

**Table 1:** Prevalence of PcV P4P by year and county council

Year	Blekinge	Dalarna	Skåne	Våsternorrland	Halland	Kronoberg	Stockholm	Sörmland
2006								
2007								
2008								
2009			X					
2010	X	X	X	X				
2011	X	X	X	X	X		X	X
2012			X	X		X	X	X
2013				X		X	X	X

Notes. X = county council uses a P4P indicator related to the PcV share. PcV share = Penicillin V prescriptions' share of total consumption of antibiotics prescribed for respiratory tract infections (ATC codes: J01CE02/J01AA02/J01CA04/J01CR02/J01DB‐DE/J01FA), children 0–6 years.PcV = penicillin V; P4P = pay‐for‐performance.

Because each county developed its own scheme, P4P targets differed between counties. Most used a target close to 80%. Stockholm county used a reference target of 70%, with bonus/penalties linearly related to deviations from this level (bonuses/penalties for higher/lower performance). Bonuses were linearly related to performance also in Kronoberg (in the range of 65–80% PcV). Another notable exception is Halland, using penalties rather than bonuses, with 69% PcV as the threshold.

We unfortunately lack information about actual payments, but back‐of‐the envelope calculations suggest that for a care center of average size, the PcV‐related P4P would account for between 0.05% and 1.2% of total reimbursement. Obviously, the impact on the personal incomes of prescribing physicians—who are typically salaried—was negligible. Though the care centers were probably aware that P4P was a minor source of revenue, it was likely difficult to estimate its size. Indeed, in the county of Skåne, the bonus size depended on the number of care centers meeting the target, rendering such calculations impossible to make. 9Some counties abolished the indicator after a few years. According to personal communication with county administrators, some of the reasons were dissatisfaction with the measure and/or a wish to prioritize other aspects of antibiotic stewardship. In Halland, the administration worried that care centers would game the system by issuing more PcV prescriptions.


#### 4.1.2 Potentially confounding policies

Simultaneously with the national PcV target in 2009, the Strama network launched targets related to the total antibiotic use and prescriptions for urinary tract infections. Some counties—e.g., some using PcV P4P—also tied P4P to these other measures. 10See online Appendix. The reason we did not evaluate the P4Ps related to urinary tract infections is that we lack outcome data for the relevant patient group (women). The main reason we do not evaluate P4P indicators for total consumption is that they were introduced simultaneously with a national incentive with the same purpose (see main text), meaning that we could only study the marginal effect of adding one incentive to another. Such an analysis would be difficult to interpret, and the policy implications are limited.Further, in 2011–2014, the national government ran a P4P program (Patientsäkerhetssatsningen) targeting the counties (i.e., not the primary care centers). The overarching goal of that program was to reduce the overall number of antibiotic prescriptions. But to receive a bonus, counties also had to institute local Strama groups that should promote antibiotic stewardship (Swedish Government, 2010).

## 5 DATA

### 5.1 Data sources

The Swedish Prescribed Drug Register comprises all purchases of prescribed pharmaceuticals since July 2005. The data are reported by the pharmacies, the only retailers of antibiotics in Sweden. Our main analysis uses yearly data on RTI antibiotics consumption of all children aged 0–6 years in 2006–2013. Further analyses use data on the consumption of all antibiotics, and some other substances, for residents of any age (Section 7.3 and Appendix A.1). The register records the patients' municipality of residence; thus, we use data aggregated at municipality level. County borders never cut across a municipality, that is, all citizens of a given municipality "belong" to the same county. We exclude one municipality (Norrtälje), where the responsibility for health care is shared by the county and municipality and for which we lack information about the reimbursement system. Our sample contains 2,312 observations: 289 municipalities in 21 county councils observed yearly from 2006 to 2013.

There are some limitations of the register: It only includes redeemed prescriptions and holds no diagnosis information. Further, the data include prescriptions from all provider types (primary care, outpatient specialist care, and dentistry). 11In specialist care, antibiotics‐related P4P is rare: We know of only one example, introduced in 2014.The register holders advised us against using the variable that indicates provider type, as they do not check its quality. However, assuming that the provider type variable gives a rough indication (fewer than 2% of prescriptions lack information on provider type), around 80% of children's RTI antibiotics are prescribed in primary care (similar in P4P and non‐P4P counties) and dentistry accounts for a negligible share. 12The information disaggregated by provider type mentioned here was provided to us by the Swedish eHealth Agency, one of the register holders, for 2011–2013. For the population as a whole, irrespective of age, estimates from other sources suggest that primary care accounts for about 50% of outpatient antibiotics (Mölstad, Erntell, & Hanberger, 2008). The difference between the estimates is reasonable, as RTI antibiotics heavily dominate children's consumption and RTI typically is treated by primary care (Section 2).


Our data also include official statistics on municipality characteristics in terms of demography, income and educational level, and county council level policy variables collected by ourselves (Appendix A.2). We also use data from the Social Insurance Agency on the number of cases of temporary parental leave due to care‐taking of sick children.

### 5.2 Descriptive statistics

**Figure 1:**
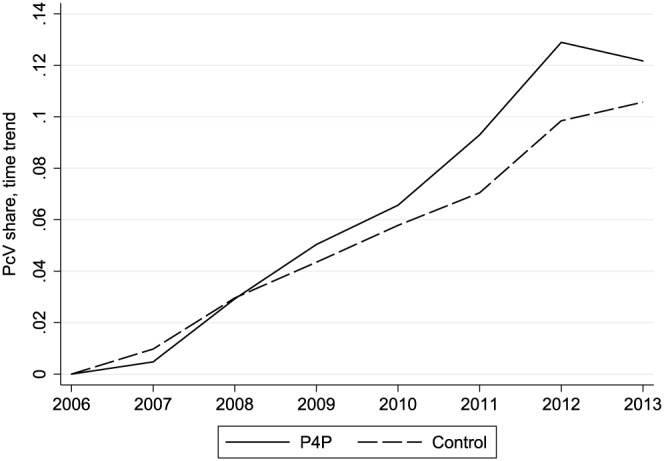
The figure shows the estimated year effects from regressions of the penicillin V (PcV) share on a vector of year dummies and municipality fixed effects. Separate estimations for municipalities in county councils that ever used pay‐for‐performance (P4P) and municipalities in county councils that never used P4P (control). The estimates are weighted by population size

Our main dependent variable is the PcV share, that is, the number of PcV prescriptions divided by the total number of RTI antibiotics prescriptions to children between 0 and 6 years of age. Appendix A.1 lists included substances. Table 2 shows the average PcV share in 2006, before any county had introduced PcV P4P, and in 2013. The group that ever used P4P had a lower PcV share at both points in time, though only the latter difference is significant. 13The additional data disaggregated by provider type (unfortunately not available before 2011, see Section 5.1 and footnote^12^) suggest that there is practically no difference between the PcV shares of primary care providers in P4P and non‐P4P counties. Instead, the difference is due to specialists in P4P counties prescribing more broad‐spectrum drugs than specialists in non‐P4P counties.The table further indicates that the PcV share increased over time in both groups. This is confirmed by Figure 1, which shows the yearly growth of the PcV share in relation to the initial sample year, again by treatment group status. The two groups experienced the same average growth between 2006 and 2008, the last year before any county introduced P4P (χ ^2^ = 2.62, p = 0.269 in joint test). Notably, before 2011, only four of the counties in the P4P group had actually implemented P4P; in fact, up to and including 2011, the trend reflects a mix of the growth in counties using P4P and counties not yet having introduced P4P (cf. Table 1). The P4P group diverged substantially from the control group in 2011, the year when the number of councils using P4P rose substantially (from 4 to 7).

**Table 2:** PcV share by treatment group

	2006		2013		
	Mean	SD	Mean	SD	Obs.
Ever P4P	0.585	0.086	0.706	0.043	109
Never P4P	0.638	0.078	0.743	0.055	181
(Control)					
Unconditional	0.612	0.086	0.724	0.053	290

Note. The first two rows show the means and standard deviations (SD) of the PcV share^a^ by year, conditional on treatment group status (ever P4P (treatment) or never P4P (control)). The third row shows the unconditional mean and SD by year. Municipality‐level data weighted by municipality population size. ^a^PcV share = penicillin V prescriptions' share of total consumption of antibiotics prescribed for respiratory tract infections (ATC codes: J01CE02/J01AA02/J01CA04/J01CR02/J01DB‐DE/J01FA), children 0–6 years. P4P = pay‐for‐performance.

**Figure 2:**
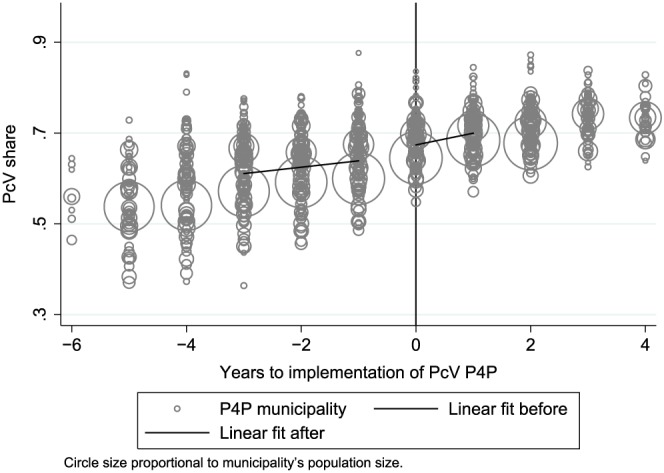
The circles show each pay‐for‐performance (P4P) municipality's penicillin V (PcV) share, plotted against the time (in years) to P4P implementation (t = 0). Circles are proportional to municipality population size

To see more clearly how the PcV share relates to the implementation of P4P, Figure 2 plots each treated municipality's PcV share in event time (i.e., t = 0 is the P4P implementation year). Each circle represents a municipality year, and the circle size is proportional to the municipality's population size. The number of municipalities at each point in (event) time vary according to the length of time we observe them before/after the implementation of P4P; for example, Stockholm, the largest circle, is observed 6 years before and 2 years after. The figure also shows two regression lines: one for the period up to t = 0, one for t = 0, and onwards. The vertical distance between the endpoint of the first line and the starting point of the second indicates that the PcV share jumps when P4P is introduced. 14The online appendix shows that the jump is attenuated if we shift back the break 1 year, suggesting that something happened at t = 0 rather than before.


## 6 EMPIRICAL STRATEGY

### 6.1 Identification issues

We use a DID strategy to estimate the P4P effect (Angrist & Pischke, 2008). The implementation of P4P was exogenous for primary care centers, which have no influence over their county's reimbursement system. But as the adoption of P4P was a choice variable for the counties, a discussion of identification issues is still warranted.

First, the decision to adopt P4P may relate to the ability to improve. Recall that the initial PcV share was relatively low in the P4P counties. This may mean that care centers in these counties could respond to the policy with relative ease, for example, if they had a large stock of patients that could be given PcV instead of broad‐spectrum antibiotics. In this case, we overestimate the effect in other contexts, though a treatment‐effect‐on‐the‐treated interpretation is still valid.

Second, the municipalities in the treatment group might have increased their PcV share more, even if the counties they belonged to had not implemented P4P. For instance, a positive effect may reflect catching‐up from a low initial level. If there are catching‐up effects, then we would expect a divergence between P4P and non‐P4P counties already before P4P was introduced. We test this formally in Section 7.3.

Third, we may be confounding the P4P effect with the effects of other policies or differential RTI morbidity. To account for underlying features and trends in individual municipalities, our specification includes municipality fixed effects and linear trends (cf. Li, Hurley, DeCicca, & Buckley, 2014). 15Unfortunately, we have too few pre‐P4P data points to apply the synthetic control method (Kreif, Grieve, Hangartner, & Turner, 2015).We also include year dummies to capture national trends. In Section 7.2, we further account for background characteristics, first by adding time‐varying covariates to the baseline specification, and second by assigning greater weight to control group observations that are similar to the P4P municipalities in terms of (a) the covariates and (b) the PcV share in 2006 (this estimation uses data for 2007–2013). We use the entropy balancing algorithm developed by Hainmueller (2012) to obtain weights. The entropy weights change yearly, as the DID strategy implies that P4P municipalities belong to the control group before they introduce P4P. In Section 7.3, we run placebo estimations to assess the probability of confounding with other policies and differential morbidity.

### 6.2 Estimation

We estimate the following equation:

(1) $$y_{mct}=\alpha \times ({\text{PcV P4P}}_{mct})+\theta_{mc}\times t+\lambda_{t}+\mu_{mc}+\varepsilon_{mct},$$

where *y*_*mct*_ is the value of the dependent variable in year *t* for municipality *m* in county council *c*. In our main estimations, *y*_*mct*_ equals the PcV share of children aged 0–6 years, but we also use the same model to consider other outcomes, for example, the number of PcV and other RTI antibiotics prescriptions for children (Sections 7 and 7.3 and Appendix A.1). *μ*_*mc*_ are municipality fixed effects and *λ*_*t*_ year fixed effects. *θ*_*mc*_ is a vector of municipality‐specific coefficients on the linear trend variable *t*. *ε*_*mct*_ is an idiosyncratic error term.

The dummy *P* *c* *V* *P*4*P*_*mct*_ equals one for observations affected by P4P, and thus, *α* captures the P4P effect. Because some counties removed P4P after a few years, we adopt two treatment definitions. First, we classify municipalities as affected by P4P in years when their county employed P4P and as unaffected otherwise. To account for the possibility that P4P had a persistent effect on prescription routines (cf. Celhay, Gertler, Giovagnoli, & Vermeersch, 2015), we then augment the specification with a dummy (*post PcV P4P*) for observations previously affected by P4P. This leads to our second treatment definition, in which *P* *c* *V* *P*4*P*_*mct*_ capture observations *currently or previously* affected by P4P.

The model is estimated with Stata module *xtivreg2* (Schaffer, 2010). Standard errors are clustered at the county level using Stata's cluster option (Rogers, 1993). Because the small number of clusters (21) may lead to underestimated standard errors (Cameron & Miller, 2015), we also subject our preferred specification to the wild cluster bootstrap with 999 replications (Cameron, Gelbach, & Miller, 2008). 16We use v.2.0.0 of *cgmwildboot*, a Stata module developed by Judson Caskey.Regressions are weighted by the population size.

Acknowledging that our main dependent variable is a fraction bounded between 0 and 1, we estimate a fractional response model as a sensitivity check. We estimate the model proposed by Papke and Wooldridge (2008), which accounts for unobserved time‐invariant features by conditioning on time‐averages of covariates instead of municipality fixed effects. We calculate the average partial effect according to the formula for nonlinear DID models in Puhani (2012) and bootstrap the average partial effect with 999 replications.

## 7 RESULTS

### 7.1 Main results

In column 1 of Table 3, municipalities are classified as treated during the years their county employs P4P. The P4P effect of 1.1 percentage points is positive and statistically significant (*p* = .008). In column 2, we add the *post PcV P4P* dummy variable. The P4P effect increases to 1.8 percentage points (*p* = .001), and the estimate on *post PcV P4P* indicates persistence. Because the two dummies are not significantly different from each other (*p* = .48), we replace them with a joint dummy for municipalities in counties that currently or previously have used P4P. Column 3 thus shows our preferred specification, in which the P4P effect is estimated at approximately 1.8 percentage points.

**Table 3:** Baseline estimations

	(1)	(2)	(3)	(4)	(5)	(6)
*y*	*PcV share*	*PcV share*	*PcV share*	*PcV*	*RTI broad*	*total*
PcV P4P	0.0106***	0.0180***	0.0176***	0.525	−0.509	0.017
	(0.00399)	(0.00562)	(0.00550)	(0.558)	(0.322)	(0.792)
post PcV P4P		0.0209**				
		(0.00822)				
Mean (*y*)	0.69	0.69	0.69	20.1	9.9	30.0
Observations	2,312	2,312	2,312	2,312	2,312	2,312
Municipalities	289	289	289	289	289	289
*R* ^2^	0.009	0.015	0.015	0.002	0.004	0.000

*Note.* The table shows baseline estimations of the effect of P4P related to the PcV share (= penicillin V prescriptions' share of total consumption of antibiotics prescribed for respiratory tract infections (ATC codes: J01CE02/J01AA02/J01CA04/J01CR02/J01DB‐DE/J01FA), children 0–6 years). In columns 1–3, the dependent variable is the PcV share. In columns 4–6, the dependent variables are the number of PcV prescriptions (col. 4; ATC code J01CE02), other RTI antibiotics prescriptions (col. 5; ATC codes: J01AA02/J01CA04/J01CR02/J01DB‐DE/J01FA), and the total of PcV and other RTI antibiotics prescriptions (col. 6) in ages 0–6 years. In columns 1 and 2, *PcV P4P* = 1 for observations in county councils that currently use P4P. In column 2, *post PcV P4P* = 1 for observations in county councils that have previously used P4P. In column 3–6, *PcV P4P* = 1 for observations in county councils that *currently use or have previously used* P4P. All specifications include municipality and year fixed effects and municipality‐specific linear trends and are weighted by the municipality population size. Standard errors clustered by county council in parentheses. PcV = penicillin V; P4P = pay‐for‐performance; RTI = respiratory tract infections. ***p<.01, **p<.05, *p<.1.

It is possible to increase the PcV share simply by prescribing more PcV. If that is the mechanism behind the increased PcV share, it is hardly consistent with antibiotic stewardship. In the next columns of the table, we change the dependent variable to the number of redeemed prescriptions of PcV (column 4) and other RTI antibiotics (column 5), respectively. Though insignificant, the estimates are of opposing sign and approximately same size. The zero net effect is confirmed in column 6, in which the dependent variable is the sum of PcV and other RTI antibiotic prescriptions. Thus, the main result reflects a substitution of PcV instead of broad‐spectrum antibiotics, exactly the behavior that the incentive was designed to stimulate.

An objection against interpreting the increasing PcV share of the treatment group as due to P4P is that it could reflect a contemporaneous drop in RTI morbidity. That is, with fewer RTI patients, there would be fewer complicated cases and thus less need for broad‐spectrum drugs. But if morbidity had decreased, we would also expect fewer prescriptions in total, contrary to what column 6 shows.

### 7.2 Sensitivity checks

Column 1 of Table 4 shows that the significant P4P effect remains when we employ the wild cluster bootstrap. 17The reason for the low bootstrap *p*‐value is that none of the 999 bootstrap samples yielded a smaller t‐statistic.The specification reported in column 2 includes covariates, leading to a slightly attenuated and less precise (*p* = .030) estimate. Of the covariate estimates, reported in the online Appendix, only one is significant (the mean personal income). Notably, the estimate on a dummy for control group municipalities using other antibiotics‐related P4P indicators (Section 4.1.2) is small and insignificant.

**Table 4:** Sensitivity

	(1)	(2)	(3)	(4)	(5)	(6)	(7)
PcV P4P	0.0176***	0.0159**	0.0212	0.0192*	0.0153**	0.0252***	0.0189
	(0.000)	(0.0073)	(0.0157)	(0.0112)	(0.0068)	(0.0063)	(0.0120)
CI lower	0.00698						
CI upper	0.0282						
Municipality FE	X	X	X	X	X	X	
Year FE	X	X	X	X	X	X	X
Municipality trends	X	X			X	X	
Covariates		X		X			X
Population weights	X	X	X	X		X	X
Observations	2,312	2,304	2,312	2,304	2,312	2,016	2,312
Municipalities	289	288	289	288	289	289	289
*R* ^2^	0.914	0.020	0.027	0.095	0.004	0.059	

*Note.* Sensitivity tests for the main dependent variable, that is, the PcV share (= penicillin V prescriptions' share of total consumption of antibiotics prescribed for respiratory tract infections (ATC codes: J01CE02/J01AA02/J01CA04/J01CR02/J01DB‐DE/J01FA), children 0–6 years; mean = 0.69). (1) Wild cluster bootstrap on the preferred specification (999 replications, *p*‐value in parenthesis; CI lower/upper = 95*%* confidence interval); (2) preferred specification + time‐varying covariates (share of children and elderly, population share with secondary/tertiary education, log population and mean income in municipality, I (control county with other antibiotic‐related P4P), I (implementation year of entry/choice reform), I (drug cost responsibility of care centers)); (3) preferred specification but excluding municipality linear trends; (4) preferred specification, excluding municipality linear trends, and including covariates; (5) preferred specification not using population size weights; (6) preferred specification using entropy balancing weights (2007–2013 sample; see the online Appendix for entropy balancing results); (7) fractional response model. Columns 2–7: standard errors clustered by county council in parentheses (cluster‐bootstrapped with 999 replications in col. 7). P4P = pay‐for‐performance; FE = fixed effects. ***p<.01, **p<.05, *p<.1.

In columns 3–4, we remove the municipality linear trends. The P4P effect increases slightly but is only significant when covariates are included (column 4). In contrast to the preferred model, the covariates thus improve precision when trends are excluded. This is reasonable: The covariates should be more important in the absence of trends, which pick up much of the variation. 18We partial out the trends in the reported estimations; otherwise R^2^ is 0.52 in our preferred specification.Column 5 shows unweighted regression results. The estimate is slightly smaller than the baseline, suggesting a stronger effect for larger municipalities. In column 6, we apply the entropy balancing weights; the P4P effect is even larger than in the baseline. 19Note that this estimation sample starts in 2007. Excluding 2006 from our baseline specification does not affect the P4P estimate. But for this reason, and because a few control municipalities receive relatively large weights, we prefer our baseline specification. This finding aligns with the smaller estimate in column 5: The balancing algorithm gives higher weight to large control municipalities, as the average population size is smaller in the control group than in the treatment group. 20The online Appendix shows the balance of the treated and entropy‐weighted control group in terms of mean, variance, and skewness. Indeed, only 5*%* of the control observations (about 100) get weights that are at least as high as the unit weight of treated observations. Finally, in column 7, we show the results from the Papke–Wooldridge fractional response model. The point estimate is slightly larger than the baseline, though it is just above the 10% significance level (*p* = .118). A disadvantage of the fractional response model is that it does not account for municipality fixed effects or linear trends.

### 7.3 Counfounding

We next address the identification threats discussed in Section 6.1. To check if the estimate picks up already existing differential trends, we include placebo dummies for the 2 years before P4P was introduced. Column 1 of Table 5 shows that the P4P effect is hardly affected and the placebo estimates are small and insignificant. Importantly, this speaks against worries that Strama's national PcV target drives the effect: The target was launched during the placebo years for seven of the eight P4P counties; thus, we would expect large placebo effects if the national target drove the effect.

**Table 5:** Counfounding checks

	(1)	(2)	(3)	(4)	(5)	(6)	(7)	(8)	(9)	(10)
*y*	*PcV share*	*D02A*	*N06AA*	*N05C*	*statin*	*PcV share*	*NonRTIAb*	*Nasal prep.*	*Cough & cold*	*Temp leave*
PcV P4P	0.0179*	1.808	−0.628	−2.415	0.193	0.0155**	−0.109	0.417	−3.999	0.827
	(0.00913)	(1.141)	(0.446)	(2.288)	(0.543)	(0.00781)	(1.822)	(0.579)	(2.794)	(2.166)
Placebo t‐1	0.000607									
	(0.00790)									
Placebo t‐2	0.000426									
	(0.00444)									
PcV P4P ×P50						0.00342				
						(0.00989)				
Mean of *y*	0.69	97.3	56.0	496.8	97.9	0.69	350.5	116.0	159.0	184.2
Observations	2,312	2,312	2,312	2,312	2,312	2,023	2,312	2,312	2,312	2,312
*R* ^2^	0.016	0.008	0.001	0.001	0.000	0.018	0.000	0.000	0.013	0.001
Municipalities	289	289	289	289	289	289	289	289	289	289

*Note.* (1) Preferred specification including two placebo year effects, that is, dummy variables for the 2 years before implementation of P4P. (2)–(5) Preferred specification with various placebo substances as dependent variables (per 1,000 residents): (2) D02A = prescriptions of emollients and protectives with no specific therapeutic effect. (3) N06AA = prescriptions of antidepressants. (4) N05C = prescriptions of sleeping pills; (4) statin = number of statin users (ATC codes: C10AA, C10BA). (6) Preferred specification including an interaction between P4P and a dummy for municipalities whose PcV share^+^ in 2006 was below the national median (2007–2013 sample). (7) Preferred specification with dependent variable = all antibiotic prescriptions except RTI^++^ antibiotic prescriptions for children aged 0–6 years (per 1,000 residents). (8)–(9) Preferred specification using other dependent variables related to RTI^++^ morbidity (per 1,000 residents): (8) prescriptions of nasal preparations (ATC R01); (9) Prescriptions of cough and cold preparations (ATC R05). (10) Cases of temporary parental leave due to care‐taking of sick children. Standard errors clustered by county council in parentheses. ^+^ PcV share = penicillin V prescriptions' share of total RTI^++^ antibiotic consumption of children 0–6 years. ^++^ RTI = respiratory tract infections. ***p<.01, **p<.05, *p<.1.

One concern is that the estimated effect picks up *other* county‐specific policy changes. For instance, recent patient choice reforms might have affected physicians' sensitivity to patients' demands. To check this, we estimate models of placebo outcomes unrelated to RTI (columns 2–5): prescriptions of emollients and protectives with no specific therapeutic effect, antidepressants, sleeping pills, and the number of statin users (see Appendix A.1 for exact definitions). The outcome variable in column 2 includes substances that can be bought over the counter, but patients may still request a prescription to reach the annual cost ceiling in the drug subsidy system. Antidepressants, sleeping pills, and statins are used by many Swedes (around 10*%* of the population, Socialstyrelsen 2016). There is no significant "treatment effect" on any of these outcomes.

Another concern is that the result may be driven by county‐level incentives tied to other antibiotics indicators (Section 4.1.2). We do not think that this is is an important issue, as the PcV share is clearly most related to the PcV‐related P4P. Also, a dummy variable for control group municipalities using P4P for other antibiotics indicators has no impact on our estimate (results not shown).

A related issue is that Strama's national targets and the national government's incentive program might have had particularly strong impact on the P4P counties. Likely, the national policies inspired the eight counties to implement antibiotics‐related P4P. Although that does not mean that the prescribers in these counties reacted differently to national policies than prescribers in other counties, we cannot fully rule it out. However, two pieces of evidence suggest that the national incentive program was of limited importance. First, one potential confounding mechanism is that the national program encouraged counties to promote antibiotic stewardship via local Strama groups, which may have focused on disseminating RTI guidelines in places where the PcV share was low. If this mechanism is the real cause of the treatment effect, it should be driven by the P4P municipalities with low initial PcV shares. That is not the case, though: When we interact the treatment variable with a dummy for municipalities whose PcV share was below the national median in 2006, the interaction is small, insignificant, and far from soaking up the baseline effect (column 6). 21Note that the estimation sample starts in 2007. However, this still does not rule out that the Strama groups might have been more effective in P4P counties than in non‐P4P counties. 22Although when asked directly, experts at the Public Health Agency of Sweden, who monitored the Strama groups' work during the national initiative, did not point out any of the P4P counties as exceptionally successful (personal communication, Nov. 21 2016).


A second indication that the P4P counties did not react strongly to the national incentive program is that their overall antibiotic consumption did not decrease more than in other counties. Reducing overall consumption was the most salient target of the national program. If P4P counties were relatively responsive to the national program, we would expect larger reductions there. 23Not least as they prescribe above the national average: in 2010, 383 prescriptions per 1,000 residents in P4P counties, 357 in control counties (*p* = .000).But we saw in column 6 of Table 3 that the total RTI antibiotic consumption of children developed similarly in P4P and non‐P4P counties, and column 7 of Table 5 shows that the same is true for the remaining part of total antibiotic consumption.

Lastly, to further check that differential RTI morbidity is not a confounder, we consider additional morbidity correlates: the number of prescriptions of nasal preparations (column 8) or cough and cold preparations (9), and the number of cases of parental work absence due to sick children (10). All estimates are insignificant.

## 8 CONCLUDING REMARKS

We find that P4P encourages primary care practices to increase the share of narrow‐spectrum antibiotics prescribed to children with RTI, and there are no signs that physicians gamed the system by issuing more prescriptions overall. As the financial incentives were small and not tied to physicians' salaries, the P4P effect was likely driven by something else than just a desire to boost income (cf. McDonald, White, & Marmor, 2009). A plausible mechanism is that P4P made the antibiotics issue more salient, thus lowering physicians' psychological barriers to changing prescription routines (Celhay et al., 2015). After having established a routine, another impetus is required to reverse it. This may explain why the effect persisted in counties that later removed the monetary incentive.

Among the limitations of the study is that it only addresses the selection of antibiotic type. Another important part of antibiotic stewardship is to limit unnecessary prescriptions. A further limitation is that the studied antibiotics may also be prescribed for other reasons than RTI. Notably, the limitation was built into the P4P systems of most of the P4P counties, which relied on the same data to follow up care center performance (one of the reasons why the targets were set far below 100*%*). To our knowledge, there were no incentives for other diagnoses that may affect the PcV share. A related limitation is that our data include prescriptions from both primary and secondary care, though primary care is the most important source of RTI prescriptions to children, and the share of RTI specialists was very similar in P4P and non‐P4P counties throughout the sample period (Socialstyrelsen, 2016). Finally, our empirical strategy does not allow us to rule out that the differences between P4P and comparison counties reflect differential reactions to national targets and incentives that were introduced during the study period. However, we show that the P4P counties did not react differently to a more salient aspect of the national incentive program to promote antibiotic stewardship (a target for the total number of antibiotics prescriptions).

Our baseline effect of 1.8 percentage points amounts to 3*%* of the pre‐P4P PcV share, or 20*%* of a standard deviation. It is similar to the improvement found in a previous study of P4P in the UK hospital sector (McDonald et al., 2015) but smaller than the effects in recent studies from China, where the overall prescription rate is considerably higher (Yip et al., 2014; Gong et al. 2016). According to the European Centre for Disease Prevention and Control, Sweden is among the European countries with the lowest overall antibiotics consumption and highest reliance on narrow‐spectrum drugs (see e.g., ECDC, 2016). Possibly, P4P yields substantial gains when applied in high‐prescribing contexts but more limited effects elsewhere. This would align with the observation that non‐financial interventions to improve antibiotic stewardship have typically had larger impact outside Scandinavia (e.g., Ranji et al., 2006). In the global fight against resistance, it is of foremost importance to find policies for high‐prescribing contexts, where resistance is higher and more likely to develop further (Goossens et al., 2005); we provide a conservative estimate for that policy discussion.

In relation to the general P4P literature, the magnitude of our estimate is similar to the typical impact of P4P for process measures in other contexts (Van Herck et al., 2010; Ogundeji et al., 2016). The result also aligns with the only previous Swedish study on P4P, which concerns diabetes‐related process measures (Ödesjö et al., 2015). Our main finding may be particularly interesting for English readers, as a similar indicator was recently introduced in the English Quality and Outcomes Framework scheme (NHS, 2016). 24The Quality and Outcomes Framework target is that the share of certain broad‐spectrum antibiotics in total prescriptions should either be less than than 10*%*, or reduced by 20*%* from the 2014/2015 value.The Swedish and English primary care systems share many features, for example, publicly financed general practitioners operating in group practices with average patient stocks of similar size (Santos, Gravelle, & Propper, 2017; Anell, 2015). However, there are also notable differences, such as the higher reliance on narrow‐spectrum drugs and larger share of publicly owned practices in Sweden. P4P also accounts for a much larger share of total reimbursement in the UK, although the importance of each P4P indicator is small in both countries.

## Supporting information

Table B.1: Other antibiotics P4P indicators in treated counties
Figure C.1: (a) Reproduction of Figure 2 in Section 5 of paper; (b) Same, but shift back regression line cut‐off one year.
Figure C.2: (a) Longer regression period; (b) Same, but shift back regression line cut‐off one year.
Figure C.3: (a) Unweighted data; (b) Excluding Stockholm county
Table D.1: Estimates on covariates
Table D.2: Covariate balance after entropy balancing

## APPENDIX A VARIABLES: DEFINITIONS AND SUMMARY STATISTICS

### A.2 Covariate Definitions

We include three covariates adjusting for confounding policies at the county council level. *o* *t* *h* *e* *r* *P*4*P*_*m**c**t*_ indicates control group observations in county councils that use at least one of the *non‐PcV* P4P indicators described in Section 4.1.2 (i.e., P4P related to either total antibiotics prescriptions or to urinary tract infection patients). *choicereform* equals one during the year when a county council implemented a reform that expanded patient choice and instituted free entry in primary care (all county councils implemented such reforms during the sample period), which in some municipalities was associated with a temporary increase in overall antibiotics use (Fogelberg, 2014). Finally, the covariate set also includes the dummy variable *cost responsibility*, which equals one if the county council has delegated the drug budget responsibility to primary care centers.

**Table A1:** Definitions of antibiotics‐related dependent variables

Variable	Description	Age group (years)
*PcV share*	Nominator: *PcV* (see below)	0–6
	Denominator: *total* (see below)	
*PcV*	*#* per 1,000 of J01CE02	0–6
*total*	*#* per 1,000 of J01CE02/J01AA02/J01CA04/J01CR02/J01DB‐DE/J01FA	0–6
*NonRTIAb*	*#* per 1,000 of J01 minus prescriptions in variable *total*,	All
*RTI broad*	*#* per 1,000 of J01AA02/J01CA04/J01CR02/J01DB‐DE/J01FA	0–6

*Note*. The table shows definitions of the antibiotics‐related dependent variables. *#* per 1,000 = number of redeemed prescriptions per 1,000 residents. *RTI* = respiratory tract infections. J01 is the category for antibiotics in the ATC (Anatomic Therapeutic Chemical) classification system. J01CE02 = phenoxymethylpenicillin (PcV), J01AA02 = doxycycline, J01CA04 = amoxicillin, J01CR02 = amoxicillin and enzyme inhibitor, J01DB‐DE cephalosporins, J01FA = macrolides. Data source: The Swedish Prescribed Drug Register.

### A.3 Summary Statistics: Covariates

**Table A2:** Definitions of other dependent variables

Variable	Description	Age group
*D02A*	*#* per 1,000 of emollients and protectives with no specific therapeutic effect	All
*N06AA*	*#* per 1,000 of antidepressants	All
*N05C*	*#* per 1,000 of sleeping pills	All
*Statin*	*#* individuals with statins (ATC C10AA, C10BA), number per capita	All
*Nasal prep.*	*#* per 1,000 of nasal preparations (ATC R01)	All
*Cough & cold*	*#* per 1,000 of cough medicine (R05)	All
*Temp leave*	*#* per 1,000 cases of temporary parental leave	All

*Note.* The table shows definitions (ATC codes/abbreviations) of the dependent variables in Tables 4 and 5. *#* per 1,000 = number of redeemed prescriptions/cases per 1,000 residents. Data source: The Swedish Prescribed Drug Register, The Social Insurance Agency (*Temp leave*).

**Table A3:** Covariate definitions

Variable	Description	Aggregation
*share children*	Share of residents 0–9 years (*%*)	m
*share elderly*	Share of residents >65 years (*%*)	m
*log(population)*	Log of population size in thousands	m
*mean income*	Average personal taxable income (thousands of SEK)	m
*share secondary edu*	Share of population (ages 16–74) with secondary education (*%*)	m
*share tertiary edu*	Share of population (ages 16–74) with tertiary education (*%*)	m
*other P4P*	**I**(Control council using other antibiotics‐related P4P (Section 4.1.2))	c
*choicereform*	**I**(Implementation year of entry/choice reform)	c
*cost responsibility*	**I**(primary care centers have budget responsibility for drug costs)	c

*Note.* The table shows definitions and aggregation level (m = municipality; c = county council) of covariates. Data source: own data collection (*other P4P, choicereform, cost responsibility*), Statistics Sweden, and Swedish Association of Local Authorities and Regions (SALAR).

**Table A4:** Summary statistics for 2008, by later P4P status

	Ever PcV P4P		Never PcV P4P		*p*‐value of
Variable	*Mean*	*SD*	*Mean*	*SD*	difference
*share children (%)*	11.6	(1.5)	10.6	(1.1)	.072
*share elderly (%)*	17.0	(3.3)	18.5	(3.2)	.178
*log(population)*	11.3	(1.3)	10.8	(1.3)	.043
*mean income*	250.9	(40.0)	230.0	(15.8)	.191
*share secondary edu (%)*	42.8	(6.4)	46.3	(5.4)	.056
*share tertiary edu (%)*	33.3	(10.6)	28.5	(9.1)	.074
*other P4P* ^*a*^	0.05	(0.226)	0	(0)	.001
*choicereform* ^*a*^	0.42	(0.50)	0.05	(0.23)	.330
*cost responsibility* ^*a*^	0.15	(0.35)	0.80	(0.40)	.000
Observations	108		181		

County‐council level variable. Summary statistics for 2008, separate for the municipalities in counties that did ("ever") or did not ("never") introduce P4P tied to the PcV share during the period 2009–2013. PcV share = penicillin V prescriptions' share of total consumption of antibiotics prescribed for respiratory tract infections (J01CE02/J01AA02/J01CA04/J01CR02/J01DB‐DE/J01FA), children 0–6 years. Column *p*‐value of difference shows *p*‐values from regressions of each variable on a dummy for PcV P4P county council, that is, the *p*‐values of the difference between the treated group (Ever PcV P4P) and the control group (Never PcV P4P). The regressions are weighted by the square root of the population size and standard errors are clustered at the county council level. The difference between P4P and control counties is not statistically significant when contrasted in *t*‐tests at the county level (*n* = 21). PcV = penicillin V; P4P = pay‐for‐performance.

## APPENDIX B ESTIMATES ON COVARIATES AND ENTROPY BALANCING RESULTS

### B.1

**Table B1:** Estimates on covariates

	(1)	(2)
*other P4P*	0.00278	0.00655
	(0.00386)	(0.00768)
*choicereform*		
	(0.00252)	(0.00368)
*cost responsibility*	0.00559	
	(0.00800)	(0.00973)
*log(population)*		
	(0.382)	(0.202)
*share children*	0.00429	0.0222***
	(0.00899)	(0.00701)
*share elderly*	0.00804	0.00679
	(0.00618)	(0.00584)
*share secondary edu*	0.00105	0.0139**
	(0.00563)	(0.00543)
*share tertiary edu*	0.00288	0.00550
	(0.00541)	(0.00799)
*mean income*	0.000920**	0.000708
	(0.000453)	(0.000595)
Observations	2,304	2,304
Municipalities	288	288

*Notes.* The table shows the parameter estimates on covariates from two specifications: column 1 (2) is the specification referred to in column 2 (4) of Table 4. Standard errors clustered by county council in parentheses. P4P = pay‐for‐performance. ****p*<0.01, ***p*<0.05, **p*<0.1.

**Table B2:** Covariate balance after entropy balancing

		P4P			Control	
	*Mean*	Variance	Skewness	*Mean*	Variance	Skewness
*PcVshare*_2006_	0.6238	0.009355	0.6173	0.6239	0.008396	0.9057
*other P4P*	0.1044	0.0937	2.588	0.1045	0.09363	2.586
*choicereform*	0.1456	0.1247	2.009	0.1456	0.1245	2.01
*cost responsibility*	0.7015	0.2099	0.8805	0.7015	0.2095	0.8806
*log(population)*	10.16	0.722	0.9165	10.16	1.087	0.3527
*share children*	11.56	3.052	0.3717	11.56	4.544	0.7567
*share elderly*	20.77	14.27	0.04682	20.77	19.27	0.1577
*share secondary edu*	47.35	33.54	1.506	47.36	41.27	1.03
*share tertiary edu*	28.12	90.09	1.334	28.12	99.88	1.159
*mean income*	245.5	1518	2.071	245.5	2127	2.251

*Note.* The table shows the covariate balance in terms of mean, variance, and skewness when control municipalities are weighted using weights from the entropy balancing algorithm (Hainmueller, 2012). All treated observations have a weight of 1. The balancing algorithm is run on the 2007–2013 sample, in order to be able to include *PcVshare*_2006_, the municipality's PcV share in 2006, among the covariates. PcV = penicillin V; P4P = pay‐for‐performance.
