# Supplementary material for: Can pay‐for‐performance to primary care providers stimulate appropriate use of antibiotics?
Source: Health Econ. 2017 Jul 7;27(1):e39–54. doi: 10.1002/hec.3535 (PMC5836891; doi:10.1002/hec.3535)
Supplement: Supplementary file 1 — Table B.1: Other antibiotics P4P indicators in treated counties Figure C.1: (a) Reproduction of Figure 2 in Section 5 of paper; (b) Same, but shift back regression line cut‐off one year. Figure C.2: (a) Longer regression period; (b) Same, but shift back regression line cut‐off one year. Figure C.3: (a) Unweighted data; (b) Excluding Stockholm county Table D.1: Estimates on covariates Table D.2: Covariate balance after entropy balancing [file HEC-27-e39-s001.pdf]

Online Appendix to Can pay-for-performance to primary care providers stimulate appropriate use of antibiotics?

Lina Maria Ellegård* Jens Dietrichson† Anders Anell‡

Contents

A Translation of Strama's national targets **2**

B Antibiotics-related P4P indicators in PcV P4P group, by year **3**

C Summary statistics: dependent variable **4**

D Estimates on covariates and entropy balancing results **6**

*Department of Economics, Lund University. E-mail: linamaria.ellegard@nek.lu.se

†SFI - The Danish National Centre for Social Research

‡Department of Business Administration, Lund University

A Translation of Strama's national targets

Strama's national targets are available in Swedish at their webpage and from the corresponding author upon request.[1] *The following is a direct translation of the document containing the targets:*

”**Strama's targets for outpatient antibiotic use**

1. Five years from now, the total prescription of antibiotics in Sweden should not be higher than 250 prescriptions per 1,000 inhabitants on a yearly basis.

The target comprises the whole ATC group J01 excluding methenamine and refers to the national level. The target is not applicable to individual health care units, but may be an indicator at the county council level.

2. Penicillin V should account for 80 percent of antibiotics for respiratory tract infections to children aged 0-6 years.

The target is not directly connected to the reason for the prescription, but is indirectly related to diagnosis as it is based on the pharmacies' sales data on typical "respiratory tract substances" (see below). Sales are measured by the number of prescriptions per 1,000 inhabitants and year.

Nominator: Prescriptions of penicillin V (J01CE02) expedited by pharmacies. All package sizes. Children 0-6 years.

Denominator: Prescriptions of amoxicillin (J01CA04), penicillin V (J01CE02), amoxicillin with clavulanic acid (J01CR02), cephalosporines (J01DB-DE) and macrolids (J01FA) expedited by pharmacies. All package sizes. Children 0-6 years.

3. Fluoroquinolones should account for no more than 10 percent of prescribed antibiotics for women (ages 18-79) with urinary tract infections”

The motivation for the PcV target is found later in the document, under the heading ”Luftvägsinfektioner”:

”**Respiratory tract antibiotics**

Antibiotics only contribute slightly to the recovery from most of the common RTIs in children. Pneumonia is the exception. The common cold and acute bronchitis in children should not be treated with antibiotics. Otitis in children above 2 years of age often does not require treatment. It is extremely rare that children require treatment for acute rhinosinusitis.

Penicillin V is the first-line drug when otitis and tonsillitis require treatment. Only a small share of the children get relapsed or complicated otitis or relapsed tonsillitis, in which case other drugs may be needed. Also for pneumonia, PcV is the first-line drug.

For these reasons, we estimate that PcV could account for more than 80 percent of all RTI antibiotics for children."

[1] http://strama.se/wp-content/uploads/2016/04/Stramas-mal-for-antibiotikaanvandningen-beskrivning.pdf (last accessed 2016-05-19)

B Antibiotics-related P4P indicators in PcV P4P group, by year

Table B.1: Other antibiotics P4P indicators in treated counties

Year	Blekinge	Dalarna	Skåne	Västernorrland	Halland	Kronoberg	Stockholm	Sörmland
2006								
2007								
2008								
2009								
2010								
2011	X		X					X
2012	X		X			X		X
2013	X		X			X		X

X = County uses P4P related to total consumption of antibiotics.

Year	Blekinge	Dalarna	Skåne	Västernorrland	Halland	Kronoberg	Stockholm (SLL)	Sörmland
2006				X				
2007				X				
2008				X				
2009	X		X		X			
2010	X	X	X	X	X			
2011	X	X	X	X			X	
2012		X	X	X		X	X	
2013				X		X	X	

X = County uses P4P related to antibiotic stewardship for women with urinary tract infection.

C Additional summary statistics: dependent variable

Figure C.1 shows that the jump between the regression lines in Figure 2 (Section 5 in the paper), which is reproduced in panel (a), is attenuated when we shift back the cut-off for the regressions one time period. Figure C.2 shows that we get similar a similar pattern when we exclude the two counties with shortest pre-/post periods (Skåne and Kronoberg) to extend the regression lines. Figure C.3 shows that the pattern and jump are similar with unweighted data, and when we remove municipalities located in the largest county council (Stockholm county) from the sample.

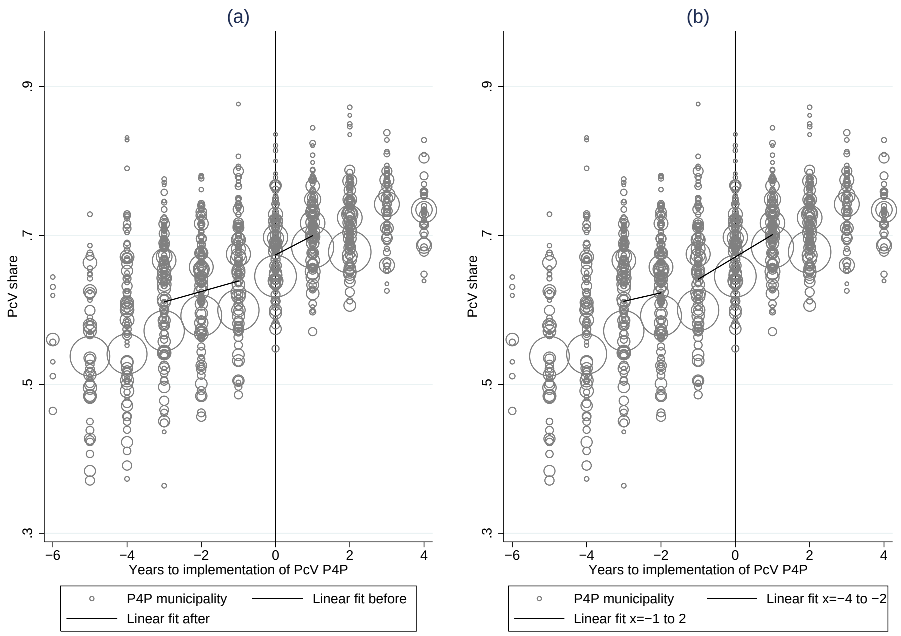

Figure C.1: (a) Reproduction of Figure 2 in Section 5 of paper; (b) Same, but shift back regression line cut-off one year.

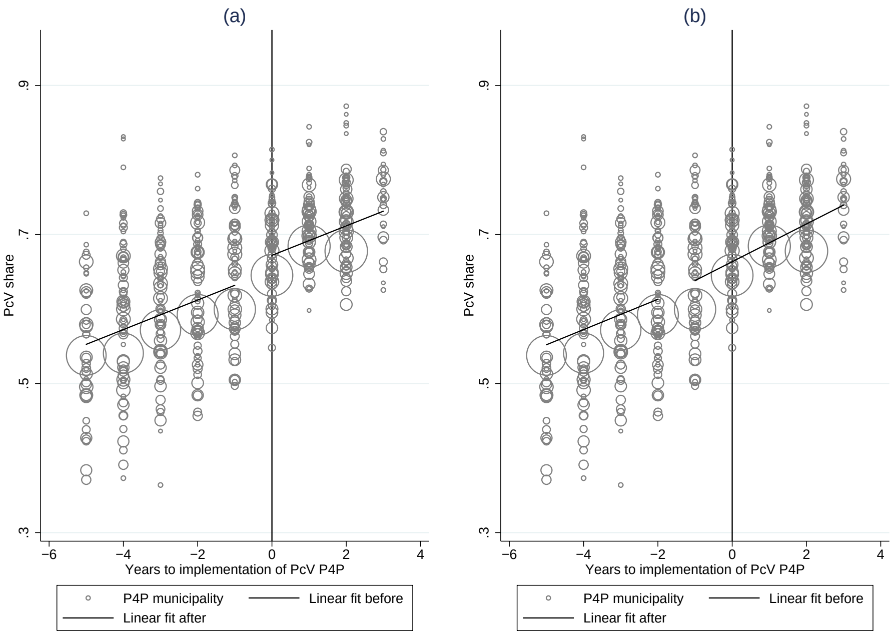

Figure C.2: (a) Longer regression period; (b) Same, but shift back regression line cut-off one year.

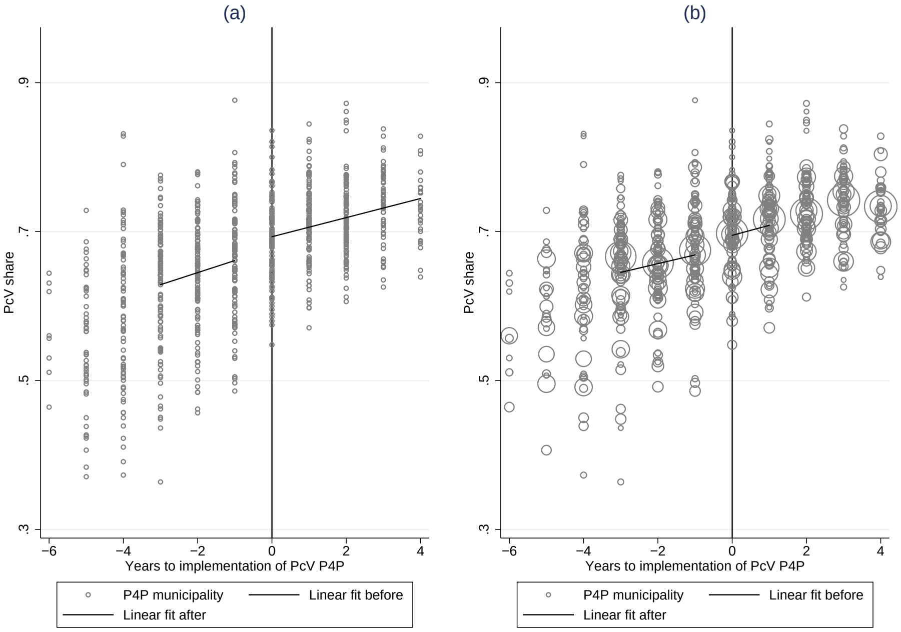

Figure C.3: (a) Unweighted data; (b) Excluding Stockholm county

D Estimates on covariates and entropy balancing results

Table D.1: Estimates on covariates

	(1)	(2)
other P4P	0.00278	0.00655
	(0.00386)	(0.00768)
choicereform	-0.000568	-0.00235
	(0.00252)	(0.00368)
cost responsibility	0.00559	-0.0122
	(0.00800)	(0.00973)
log(population)	-0.0359	-0.0195
	(0.382)	(0.202)
share children	0.00429	-0.0222***
	(0.00899)	(0.00701)
share elderly	-0.00804	-0.00679
	(0.00618)	(0.00584)
share secondary edu	-0.00105	-0.0139**
	(0.00563)	(0.00543)
share tertiary edu	0.00288	-0.00550
	(0.00541)	(0.00799)
mean income	-0.000920**	0.000708
	(0.000453)	(0.000595)
Observations	2,304	2,304
Municipalities	288	288

Notes: The table shows the parameter estimates on covariates from two specifications: column 1 (2) is the specification referred to in column 2 (4) of Table IV. Standard errors clustered by county council in parentheses. *** p<0.01, ** p<0.05, * p<0.1

Table D.2: Covariate balance after entropy balancing

	P4P			Control		
	mean	variance	skewness	mean	variance	skewness
$PcVshare_{2006}$	0.6238	0.009355	-0.6173	0.6239	0.008396	-0.9057
other P4P	0.1044	0.0937	2.588	0.1045	0.09363	2.586
choicereform	0.1456	0.1247	2.009	0.1456	0.1245	2.01
cost responsibility	0.7015	0.2099	-0.8805	0.7015	0.2095	-0.8806
log(population)	10.16	0.722	0.9165	10.16	1.087	0.3527
share children	11.56	3.052	0.3717	11.56	4.544	0.7567
share elderly	20.77	14.27	0.04682	20.77	19.27	0.1577
share secondary edu	47.35	33.54	-1.506	47.36	41.27	-1.03
share tertiary edu	28.12	90.09	1.334	28.12	99.88	1.159
mean income	245.5	1518	2.071	245.5	2127	2.251

The table shows the covariate balance in terms of mean, variance and skewness when control municipalities are weighted using weights from the entropy balancing algorithm (Hainmueller, 2012). All treated observations have a weight of 1. The balancing algorithm is run on the 2007-2013 sample, in order to be able to include $PcVshare_{2006}$, the municipality's PcV share in 2006, among the covariates.

References

Hainmueller, J., 2012. Entropy balancing for causal effects: A multivariate reweighting method to produce balanced samples in observational studies. Political Analysis 20 (1), 25–46.
